# A MAP3k1 SNP Predicts Survival of Gastric Cancer in a Chinese Population

**DOI:** 10.1371/journal.pone.0096083

**Published:** 2014-04-23

**Authors:** Xiaowei Wei, Enke Zhang, Chun Wang, Dongying Gu, Lili Shen, Meilin Wang, Zhi Xu, Weida Gong, Cuiju Tang, Jinglong Gao, Jinfei Chen, Zhengdong Zhang

**Affiliations:** 1 Department of Oncology, Nanjing First Hospital, Nanjing Medical University, Nanjing, Jiangsu Province, China; 2 Department of Environmental Genomics, Jiangsu Key Lab of Cancer Biomarkers, Prevention and Treatment, Cancer Center, Nanjing Medical University, Nanjing, Jiangsu Province, China; 3 Department of Genetic Toxicology, the Key Laboratory of Modern Toxicology of Ministry of Education, School of Public Health, Nanjing Medical University, Nanjing, Jiangsu Province, China; 4 Central Laboratory, Shanxi People’s Hospital, Xi’an, Shanxi Province, China; 5 Department of General Surgery, Yixing Tumor Hospital, Yixing, Jiangsu Province, China; Kaohsiung Chang Gung Memorial Hospital, Taiwan

## Abstract

**Objectives:**

Genome-wide association studies (GWAS) have demonstrated that the single nucleotide polymorphism (SNP) *MAP3K1 rs889312* is a genetic susceptibility marker significantly associated with a risk of hormone-related tumors such as breast cancer. Considering steroid hormone-mediated signaling pathways have an important role in the progression of gastric cancer, we hypothesized that *MAP3K1 rs889312* may be associated with survival outcomes in gastric cancer. The purpose of this study was to test this hypothesis.

**Methods:**

We genotyped *MAP3K1 rs889312* using TaqMan in 884 gastric cancer patients who received subtotal or total gastrectomy. Kaplan-Meier survival analysis and Cox proportional hazard regression were used to analyze the association between *MAP3K1 rs889312* genotypes and survival outcomes of gastric cancer.

**Results:**

Our findings reveal that the *rs889312* heterozygous AC genotype was significantly associated with an increased rate of mortality among patients with diffuse-type gastric cancer (log-rank *P* = 0.028 for AC versus AA/CC, hazard ratio [HR] = 1.32, 95% confidence interval [CI] = 1.03–1.69), compared to those carrying the homozygous variant genotypes (AA/CC). Additionally, univariate and multivariate Cox regression analysis demonstrate that *rs889312* polymorphism was an independent risk factor for poor survival in these patients.

**Conclusions:**

In conclusion, we demonstrate that *MAP3K1 rs889312* is closely correlated with outcome among diffuse-type gastric cancer. This raises the possibility for *rs889312* polymorphisms to be used as an independent indicator for predicting the prognosis of diffuse-type gastric cancer within the Chinese population.

## Introduction

Gastric cancer is one of the most common cancers worldwide, accounting for about 8% of new cancers and 10% of cancer deaths [1]. Despite improvements in diagnosis, surgery, chemotherapy, and targeted therapy, overall survival (OS) for patients with advanced stages of gastric cancer still remains poor [2]. Surgical tumor resection has been considered a primary curative treatment option for this disease, but it achieves a very poor 5-year survival rate, ranging between 20–25% in patients with advanced cancer stages [3]. Tumor staging, as the best available clinical measure, has been widely used to assess the aggression and prognosis of gastric cancer. In recent years, however, more and more investigators believe that tumor staging alone is inadequate for predicting the risk and prognosis of disease because there are important differences even within tumors of the same stage [4]. Therefore, there is a definite need to develop a novel molecular signature that can be applied as a reliable prognostic marker for gastric cancer and used in combination with traditional diagnostic and staging measures. Recently, researchers have focused on exploring genetic variants that are associated with gastric cancer development and progression [5].

Mitogen-Activated Protein Kinase Kinase Kinase 1 (MAP3K1), a serine/threonine kinase, acts in the mitogen-activated protein kinase (MAPK) pathway that involves Ras, Raf, Mek, and Erk [6], [7]. The most apparent function of MAP3K1 is to phosphorylate and activate MAPK kinase (MAPK2), which in turn phosphorylates MAPK/ERK to produce downstream signaling effects on a variety of cancer genes [8]. In 2007, Easton et al. first identified that the *rs889312* single nucleotide polymorphism (SNP), which lies in a linkage disequilibrium block of approximately 280**kb containing the *MAP3K1* gene, was a susceptibility loci for breast cancer (BC) [9]. To date, several studies have demonstrated the association between the *MAP3K1* gene *rs889312* polymorphism and the risk of BC [10]–[13]. For example, Garcia-Closas et al. have found that MAP3K1 variants were relevant in estrogen receptor (ER)-positive tumors to a greater degree than in ER-negative tumors [13]. Moreover, the *MAP3K1 rs889312* variant genotype was found to be associated with larger breast tumors in Asians but not in European populations [12].

Since the expression of sex hormone receptors in human gastric cancer was first reported by Tokunaga et al. [14], numerous investigators have believed gastric cancer to be a hormone-related tumor, in which steroid hormone-mediated signaling pathway plays an important role in carcinogenic progression [15]. Considering the role of the *MAP3K1 rs889312* SNP involved in the risk of BC, we therefore hypothesized that *MAP3K1 rs889312* may also be associated with survival outcomes in gastric cancer, and thus may have the potential to serve as a prognostic marker for this disease.

## Materials and Methods

### Study Population

A total of 1022 newly-diagnosed gastric cancer patients who received subtotal or total gastrectomy were recruited in Yixing People’s Hospital (Yixing, China) between January 1999 and December 2006 for this study. No patients had received chemotherapy or radiotherapy at any point prior to surgery. Written informed consent was obtained from each patient before sample collection. The protocol of this study was approved by the Institutional Review Board of Nanjing Medical University (Nanjing, China).

### Outcomes Collection

Patient follow-up was performed by telephone call every three months. OS served as the end point in this study. Date of death was obtained from inpatient and outpatient records or patients’ families through follow-up telephone calls. Survival time was calculated as the time from the date of surgery to the date of death or the end of follow-up. Patients alive on the last follow-up date were considered as censored. Clinical and pathological variables including age, gender, tumor location, differentiation, tumor size, depth of invasion, lymph node metastasis, and distant metastasis were obtained from inpatient and histopathological records. The stage of gastric cancer was evaluated according to the tumor node metastasis (TNM) classification system published by the American Joint Committee on Cancer (AJCC) in 2010 (AJCC Cancer Staging Manual, Seventh Edition). Lauren’s criteria were used to classify the tumors into intestinal-type or diffuse-type [16].

### Genotyping

Genomic DNA of patients was extracted from paraffin sections of tissues. The SNP genotyping of MAP3K1 was performed with the TaqMan SNP Genotyping Assay using the 384-well ABI 7900HT Real-time PCR System (Applied Biosystems, Foster City, CA, USA). The fluorescence profile was read on an ABI Prism 7900HT instrument and the results were analyzed using Sequence Detection Software 2.2 (Applied Biosciences, Foster City, CA, USA). Genotype analysis was carried out by two independent researchers in a blind manner. Ten percent of the samples were randomly selected for repeated genotyping in order to verify genotyping and sample-handling accuracy, and the results were 100% concordant. Nevertheless, 27 samples failed to be genotyped because of poor DNA quality. The donors of these samples were then excluded from further analysis. As a result, 884 gastric cancer patients were included in the final analysis.

### Statistical Analysis

All statistical analyses were performed using SAS 9.1 software (SAS Institute, Cary, NC, USA) with a two-sided test. The associations between OS and demographic characteristics, clinical features, and genetic SNPs were estimated using the Kaplan-Meier method and log-rank test. Mean OS was presented when the median OS could not be calculated. Univariate and multivariate Cox proportional hazards regression models were performed to estimate crude hazard ratios (HRs), adjusted HRs, and their 95% confidence intervals (CIs). The effects of genetic SNPs on survival were also evaluated using multivariate Cox regression models, which adjusted for sex, age, tumor location, differentiation, tumor size, depth of invasion, lymph node metastasis, distant metastasis, and TNM stage. A *P* value<0.05 was considered sufficient for statistical significance. The log-rank *P* values less than 0.05 were corrected by a permutation test (n = 5000 random permutations) reported by Vasselli et al [17].

## Results

### Characteristics of Patients

Of the recruited patients, 78 (7.6%) were excluded because of lack of adequate follow-up information; 33 (3.2%) were excluded because of non-gastric cancer-related deaths, such as suicide and traffic accident. Finally, a total of 884 gastric cancer patients were included in this study. All patients received surgical resections. Their baseline characteristics and clinical features are presented in Table 1. The median age of patients was 62 years (range: 28–83 years), and there were 677 males (76.6%) and 207 females (23.4%). The duration of follow-up ranged from 1.0 to 119.0 months with a median follow-up of 35.0 months (last follow-up in March 2009). During a follow-up period, 406 patients died and their causes of death were all gastric cancer-related. Survival analysis shows that tumor size, TNM stage, depth of invasion, lymph node metastasis, and histological types were significantly correlated with survival time of patients (log-rank *P*<0.05). Patients with tumor size >5 cm, or with higher TNM stage, depth of invasion, and lymph node metastasis were at a dramatically higher risk of death compared to those with tumor size ≤5 cm, or with lower pathological stage, depth of invasion, and lymph node metastasis. Additionally, patients with diffuse-type gastric cancer had a 42% increase in risk of death compared with intestinal-type gastric cancer patients (HR = 1.42, 95% CI = 1.16–1.72).

**Table 1:** Baseline characteristics and clinical features of patients.

Variables	No. of patients, n (%)N = 884	No. of deaths, nN = 406	Median OS(months)	Log-rank *P*	HR (95% CI)
***Age***					
≤60 years	416 (47.1%)	188	97.0	0.325	1.00
>60 years	468 (52.9%)	218	62.0		1.10 (0.91–1.34)
***Sex***					
Male	677 (76.6%)	307	74.0	0.381	1.00
Female	207 (23.4%)	99	63.0		0.90 (0.71–1.14)
***Tumor location***					
Cardia	102 (11.5%)	51	78.0	0.707	1.00
Body	751 (85.0%)	338	70.0		1.09 (0.82–1.45)
Fundus/pylorus	31 (3.5%)	17	29.0		1.27 (0.72–2.34)
***Differentiation***					
Poorly differentiated	501 (56.7%)	247	58.0	0.108	1.00
Moderately/well differentiated	300 (33.9%)	126	78.0		0.85 (0.69–1.05)
Unknown	83 (9.4%)	33	71.8*		0.73 (0.54–1.04)
***Tumor size***					
≤5 cm	556 (62.9%)	234	73.9*	0.0008	1.00
>5 cm	328 (37.1%)	172	51.0		1.39 (1.16–1.75)
***Histological types***					
Intestinal	376 (42.5%)	146	76.8*	0.0024	1.00
Diffuse	505 (57.1%)	258	54.0		1.42 (1.16–1.72)
Others	3 (0.4%)	2	33.0		1.91 (0.37–17.1)
***Depth of invasion***					
T1	174 (19.7%)	57	84.3*	0.0003	1.00
T2	129 (14.6%)	55	78.0		1.38 (0.95–2.03)
T3	6 (0.7%)	3	70.0		1.48 (0.40–6.43)
T4	575 (65.0%)	291	51.0		1.81 (1.32–2.13)
***Lymph node metastasis***					
N0	357 (40.4%)	128	80.4*	<0.0001	1.00
N1/N2/N3	527 (59.6%)	278	47.0		1.71 (1.38–2.04)
***Distant metastasis***					
M0	874 (98.9%)	400	74.0	0.119	1.00
M1	10 (1.1%)	6	33.0		1.87 (0.80–7.18)
***TNM stage***					
I	235 (26.6%)	80	82.7*	<0.0001	1.00
II	195 (22.1%)	78	70.8*		1.22 (0.90–1.68)
III	444 (50.2%)	242	41.0		1.91 (1.46–2.28)
IV	10 (1.1%)	6	33.0		2.60 (1.31–17.0)

OS: overall survival; HR: hazard ratio; CI: confidence interval.

*Mean OS was presented when median OS could not be calculated.

### Effects of *MAP3K1 rs889312* on Survival of Gastric Cancer Patients

**Figure 1:**
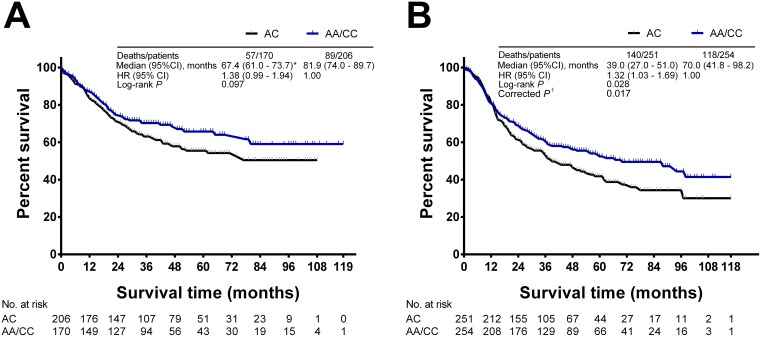
Association of *MAP3K1 rs889312* genotypes with OS in gastric cancer patients. (A) In intestinal-type gastric cancer patients, the heterozygous variant genotype (AC) did not show significant association with survival outcomes; (B) In diffuse-type gastric cancer patients, individuals with the AC genotype had a significantly increased risk of death when compared with those carrying the homozygous variant genotypes (AA/CC). HR and 95% CIs were estimated using multivariate Cox regression model. Statistical differences between two survival curves were assessed by the log-rank test. Asterisks (*) indicates mean OS when median OS could not be calculated. ^†^The log-rank *P* value less than 0.05 was corrected by a permutation test (n = 5000 random permutations) reported by Vasselli et al [17].

We performed Cox regression analyses to assess the association of *MAP3K1 rs889312* genotypes on survival of gastric cancer patients in various genetic models. The results are shown in Table 2. In the overall patient cohort, we found that there was a significant association between the genotypes of *MAP3K1 rs889312* and survival of gastric cancer patients in codominant and overdominant models (log-rank *P* = 0.046 and corrected *P* = 0.032 for codominiant model; log-rank *P* = 0.014 and corrected *P* = 0.026 for overdominant model). Results of the overdominant model reveal that the *MAP3K1 rs889312* AC variant genotype was associated with a statistically significant 30% decrease in survival compared to the AA/CC homozygotes (HR = 1.30, 95% CI = 1.06–1.58). Then, we stratified the patients by histology (intestinal-type and diffuse-type) and evaluated the association of *MAP3K1 rs889312* genotypes with survival in the stratified patients. In intestinal-type gastric cancer patients, there was no significant association between genotypes and survival in any genetic models, including an overdominant model (Figure 1A). In the patients with diffuse-type gastric cancer, however, individuals with the *rs889312* heterozygous variant genotype (AC) had a significantly increased risk of death when compared with those carrying the homozygous variant genotypes (AA/CC) (Figure 1B, log-rank *P* = 0.028, corrected *P* = 0.017, HR = 1.32, 95% CI = 1.03–1.69).

**Table 2:** Association between *MAP3K1 rs889312* polymorphism and survival outcomes of gastric cancer patients in various genetic models.

Genotype Models		Overall Patients, N = 884					
		n (%)	Deaths	Median OS	Log-rank *P*	Corrected *P* $	HR (95% CI)†
Codominant	AA	225 (25.5)	95	94.0	0.046	0.032	1.00
	AC	459 (51.9)	230	52.0			1.30 (1.01–1.68)
	CC	200 (22.6)	81	74.8*			1.04 (0.77–1.40)
Dominant	AA	225 (25.5)	95	94.0	0.196	–	1.00
	AC/CC	659 (74.5)	311	62.0			1.18 (0.94–1.49)
Recessive	AA/AC	684 (77.4)	325	62.0	0.116	–	1.00
	CC	200 (22.6)	81	74.8*			0.82 (0.65–1.05)
Overdominant	AA/CC	425 (48.1)	176	98.0	0.014	0.026	1.00
	AC	459 (51.9)	230	52.0			1.30 (1.06–1.58)
Additive	C allele						0.98 (0.81–1.19)
	Trend *P*						0.840
		**Intestinal-type Patients, N = 376**					
Codominant	AA	91 (24.2)	29	83.8*	0.221	–	1.00
	AC	206 (54.8)	89	67.4*			1.25 (0.82–1.91)
	CC	79 (21.0)	28	75.7*			0.82 (0.49–1.39)
Dominant	AA	91 (24.2)	29	83.8*	0.150	–	1.00
	AC/CC	285 (75.8)	117	71.0			1.43 (0.95–2.17)
Recessive	AA/AC	297 (79.0)	118	76.1*	0.617	–	1.00
	CC	79 (21.0)	28	75.7*			0.90 (0.60–1.36)
Overdominant	AA/CC	170 (45.2)	57	81.9*	0.097	–	1.00
	AC	206 (54.8)	89	67.4*			1.38 (0.99–1.94)
Additive	C allele						1.13 (0.83–1.56)
	Trend *P*						0.440
		**Diffuse-type Patients, N = 505**					
Codominant	AA	133 (26.3)	65	67.0	0.072	–	1.00
	AC	251 (49.7)	140	39.0			1.40 (1.01–1.92)
	CC	121 (24.0)	53	71.9*			1.12 (0.78–1.61)
Dominant	AA	133 (26.3)	65	67.0	0.466	–	1.00
	AC/CC	372 (73.3)	193	48.0			1.12 (0.84–1.49)
Recessive	AA/AC	384 (76.0)	205	48.0	0.076	–	1.00
	CC	121 (24.0)	53	71.9*			0.77 (0.57–1.05)
Overdominant	AA/CC	254 (50.3)	118	70.0	0.028	0.017	1.00
	AC	251 (49.7)	140	39.0			1.32 (1.03–1.69)
Additive	C allele						0.91 (0.71–1.17)
	Trend *P*						0.470

OS: overall survival; HR: hazard ratio; CI: confidence interval.

*Mean OS was presented when median survival could not be calculated.

$ The log-rank *P* values less than 0.05 were corrected by a permutation test (n = 5000 random permutations).

† Adjusted for sex, age, differentiation status, and TNM stage.

### Stratified Analysis of Patients with Diffuse-type Gastric Cancer

**Figure 2:**
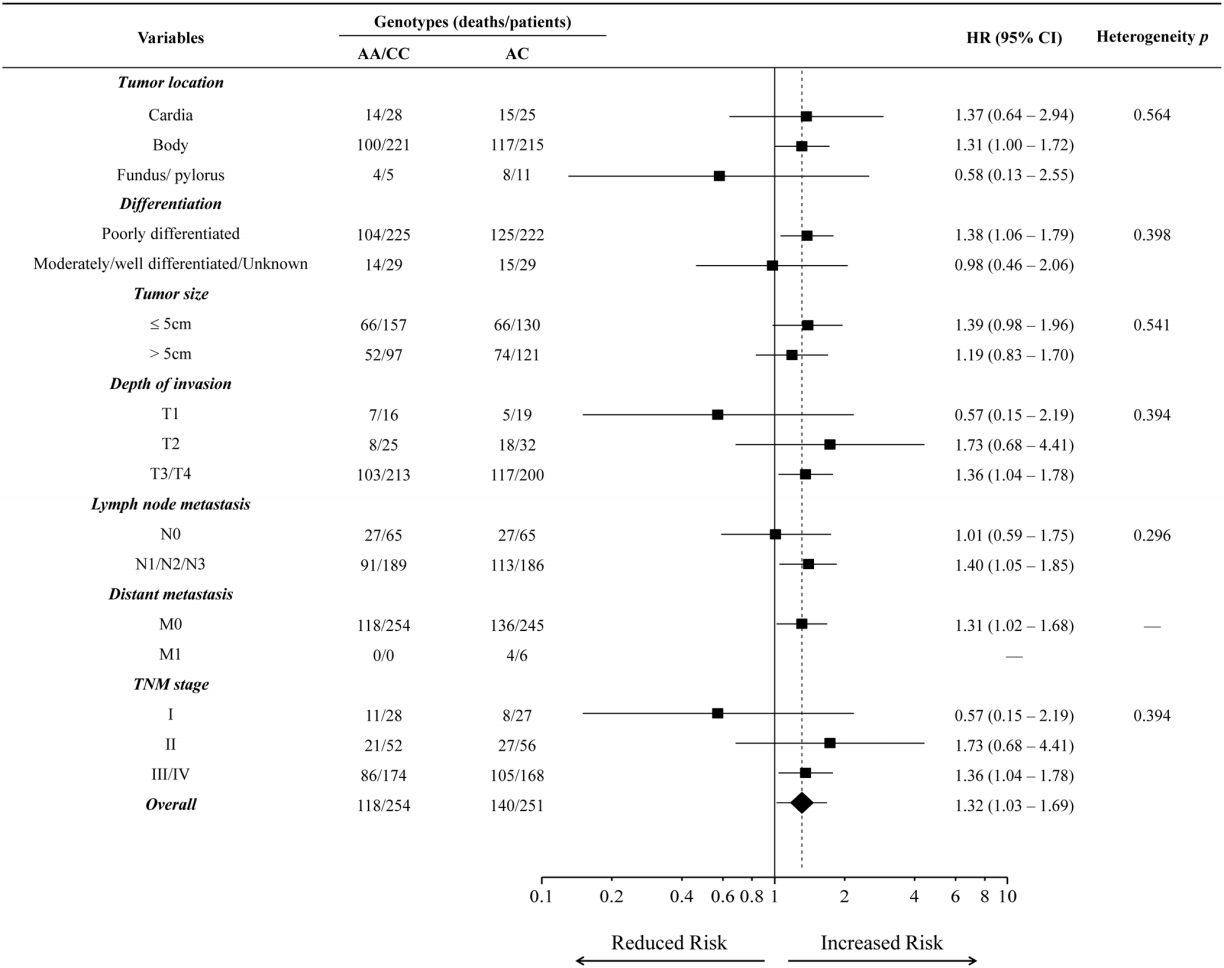
Stratified analysis of *MAP3K1 rs889312* genotypes associated with survival outcomes in diffuse-type gastric cancer patients.

The associations between *MAP3K1 rs889312* genotypes and survival of individuals with diffuse-type gastric cancer were further assessed by stratified analysis based on tumor location, differentiation, tumor size, depth of invasion, lymph node metastasis, distant metastasis, and TNM stage. As shown in Figure 2, there was not significantly differentiated association between *MAP3K1 rs889312* genotypes and diffuse-type gastric cancer survival in the different subgroups of patients. Nevertheless, there was a trend toward a higher relative risk of death associated with *rs889312* heterozygous variant genotype (AC) among patients with T2 depth of invasion and TNM stage II (HR = 1.73, 95% CI = 0.68–4.41).

### Cox Regression Analysis for Survival of Patients with Diffuse-type Gastric Cancer

We performed univariate and multivariate Cox regression analysis to explore the prognostic value of *MAP3K1 rs889312* genotypes for survival in patients with diffuse-type gastric cancer, summarized in Table 3. Univariate analysis demonstrated that *rs889312* polymorphism was a potential prognostic factor for survival among diffuse-type gastric cancer patients (*P* = 0.030, HR = 1.31 for AC vs. AA/CC). Multivariate analysis with two different models further confirmed the findings achieved in univariate analysis, indicating heterozygous AC genotype was an independent risk factor for poor survival in patients with diffuse-type gastric cancer (HR = 1.32 and 1.34 for AC vs. AA/CC, respectively).

**Table 3:** Cox regression analysis of survival in patients with diffuse-type gastric cancer.

MAP3K1		HR (95% CI)					
		Univariate analysis	*P* value	Multivariate analysis - Model 1^a^	*p* value	Multivariate analysis - Model 2^b^	*P* value
**Genotypes**	**AA/CC**	1.00	–	1.00	–	1.00	–
	**AC**	1.31 (1.02–1.68)	0.030	1.32 (1.03–1.69)	0.028	1.34 (1.04–1.71)	0.021

a Adjusted for sex, age, TNM stage, and differentiation status.

b Adjusted for all covariates including sex, age, tumor location, differentiation, tumor size, depth of invasion, lymph node metastasis, distant metastasis, and TNM stage.

HR: hazard ratio; CI: confidence interval.

## Discussion

In this study, we systematically investigated the association between the *MAP3K1 rs889312* SNP and overall survival in a relatively large population of Chinese gastric cancer patients. Our findings reveal that the *rs889312* heterozygous AC genotype was significantly associated with increased risk of mortality among patients with diffuse-type gastric cancer, compared to those carrying the homozygous variant genotypes (AA/CC). Additionally, univariate and multivariate Cox regression analysis demonstrates that the *rs889312* polymorphism was an independent risk factor for poor survival in these patients. To our knowledge, this is the first report describing the association between *MAP3K1 rs889312* polymorphism and gastric cancer survival.

The *MAP3K1* gene encodes MAP3K1, a serine/threonine kinase protein, which is involved in the MAPK signaling pathway and plays a pivotal role in regulating transcription of important cancer genes [6]. Recently, several studies have demonstrated that certain *MAP3K1 rs889312* genotypes are genetic susceptibility markers for hormone-related tumors such as breast cancer and are significantly associated with risk of these tumors [18], [19]. Jara et al. reported that the MAP3K1 *rs889312* AC genotype had a significant association with the increased risk in Chilean patients of familial breast cancer and early-onset non-familial breast cancer [11]. Rebbeck et al. confirmed the role of the *MAP3K1 rs889312* SNP in breast cancer susceptibility in African-American patients, and revealed that *MAP3K1* genotypes conferred their effects in primarily ER^+^ and PR^+^ tumors, and that patients carrying the AC or CC genotypes had a noticeably higher breast cancer risk than those carrying the AA genotype [10]. In line with these findings, the results of our study indicate that genetic variants of *MAP3K1 rs889312* were also genetic susceptibility markers for gastric cancer, in particular diffuse-type, which may be attributed to the possibility that MAP3K1 might affect gastric cancer etiology in a steroid hormone-dependent manner.

Previous studies have suggested that the steroid hormone-mediated signaling pathway has an important role in the progression of gastric cancer [20]–[22]. Of the hormones implicated in relating to gastric cancer, estrogen was reported to be closely involved in gastric cancer carcinogenesis and development. *In vitro* studies have found that estrogen could stimulate the growth of gastric cancer cell lines [23], [24]. In addition, there is clinical evidence that high levels of estrogen during pregnancy or delivery might accelerate the growth of gastric cancer [25]. These findings suggest estrogen exposure may influence gastric cancer risk. In regard to MAP3K1, this enzyme has a pivotal function in the MAPK signaling cascade implicated in the cellular response to mitogenic and metabolic stimuli, including estrogen [8], [26]. The polymorphism of *MAP3K1 rs889312* may provide a source of genomic variability in the MAPK signaling pathway. Therefore, it is biologically plausible that estrogen exposure in combination with genomic variability of MAPK cascade may influence gastric cancer susceptibility.

In this study, the SNP *MAP3K1 rs889312* was significantly associated with survival outcomes in patients with diffuse-type gastric cancer; however, this was not the case in individuals with intestinal-type cancer. This result may be attributed to the difference in the expression of estrogen receptors (ERs) among the histological types of gastric cancer. The biological actions of estrogen are mediated through two specific receptors, ERα and ERβ, which belong to the nuclear receptor superfamily. In gastric cancer, ERα expression is higher in the diffuse type than in the intestinal type [27], [28], thus increasing the susceptibility of diffuse-type tumors to estrogen exposure. Furthermore, the carcinogenesis of intestinal-type gastric cancer develops through several cascade stages with *Helicobacter pylori* infection, followed by chronic gastritis, gastric atrophy, and intestinal metaplasia [29]; whereas the diffuse-type is considered to be derived from hyperplastic stem or precursor cells and usually associated with pan-gastritis without atrophy [30]. The biological differences between these two histological types may also be responsible for this observed histology specific difference. Nevertheless, the exact underlying cause remains to be elucidated.

In a previous meta-analysis based on breast cancer, the *MAP3K1 rs889312* allele C has been identified as a low-penetrant risk factor for developing breast cancer [18]. In the current study, under the codominant model, we found that patients carrying the *MAP3K1 rs889312* CC genotype showed a slightly poorer survival than those carrying AA genotype in all the patients as well as in individual histological subtypes. However, patients with AC genotype had a much poorer survival compared with those with AA genotype, suggesting this heterozygous genotype may be a significant risk factor affecting the survival of patients. To test this assumption, we used the over-dominant model, where the homozygotes (AA/CC) have the same expected impact on survival, but the heterozygote (AC) has a different expected impact on survival. Under this model, the AC genotype showed a significant association with poor survival outcomes, which was also demonstrated by permutation tests. From a biological point of view, the SNP *rs889312* is located in the region containing the MAP3K1 gene. The AC genotype may alter the translation of the MAP3K1 gene and increase the expression of translated protein, consequently activating the MAPK signaling pathway to enhance the growth and proliferation of malignant cells. However, the exact mechanisms are largely unknown and need further investigation to clarify. Interestingly, stratified analysis showed that the association between the *rs889312* AC genotype and diffuse-type gastric cancer was more predominant among patients with T2 depth of invasion and TNM stage II. Univariate and multivariate Cox regression analysis also demonstrated that the *rs889312* AC genotype can serve as an independent prognostic factor in this type of gastric cancer. Taken together, these results demonstrate the predictive value of *rs889312* polymorphism for survival prognosis in patients with early stage diffuse-type gastric cancer.

Several limitations in our study should be acknowledged. First, we were unable to collect progression-free survival data from all patients and hence did not explore the association between *MAP3K1 rs889312* polymorphism and disease recurrence. Second, we did not investigate the association between genetic variations in *MAP3K1 rs889312* and patients’ responses to anti-cancer drugs, primarily because different chemotherapies were employed in our patients which results in a lack of statistical power to detect responses to drug effects. Third, although the maximum follow-up time was 119 months in our study, the median follow-up time was relatively short (35.0 months) because over 40% patients were recruited in the time period spanning from 2005 to 2006. Currently, we are conducting a larger prospective clinical trial with a longer follow-up time to validate our findings.

In conclusion, we have demonstrated here that *MAP3K1 rs889312* is closely correlated with survival among diffuse-type gastric cancer. This raises the possibility for *rs889312* polymorphism to be used as an independent indicator for predicting the prognosis of diffuse-type gastric cancer in Chinese population. Further validation in other ethnicity/race populations is needed, as is investigation into the mechanism of why this polymorphism would be associated with the outcome of certain steroid-hormone related cancers.
